# Caregiver strain and symptoms of depression among principal caregivers of patients with schizophrenia and bipolar affective disorder in Sri Lanka

**DOI:** 10.1186/1752-4458-7-2

**Published:** 2013-01-09

**Authors:** Chaturaka Rodrigo, Tharanga Fernando, Senaka Rajapakse, Varuni De Silva, Raveen Hanwella

**Affiliations:** 1 Department of Clinical Medicine, Faculty of Medicine, University of Colombo, Colombo, Sri lanka; 2 Department of Psychological Medicine, Faculty of Medicine, University of Colombo, Colombo, Sri lanka

## Abstract

**Introduction:**

Data on caregiver strain and depression of principal caregivers of patients with mental illnesses are few in developing countries. Findings from developed countries cannot be applied directly to developing countries as culture specific factors may influence the outcome.

**Methods:**

A prospective study was carried out in the University Psychiatry Unit of the National Hospital of Sri Lanka (NHSL) to identify symptoms of depression, caregiver strain and dissatisfaction with life in caregivers of patients with schizophrenia and bipolar affective disorder. Participants were assessed using the Center for Epidemiological Studies – Depression Scale, Satisfaction with Life Scale and the Modified Caregiver Strain Index.

**Results and discussion:**

Eighty caregivers were interviewed (males; 36, 45%). Symptoms of depression were significant in 37.5%, while 48.8% had unsatisfactory scores on the Satisfaction with Life Scale. Depression and higher caregiver strain were associated with spending more time with the patient, interruption to work, disputes with relations, being assaulted by patient and self admission of needing professional help to overcome mental stress.

**Conclusion:**

This study identified several associations for depression and increased caregiver strain among caregivers in a subset of patients with mental disorder in Sri Lanka. These can be used as markers to screen and increase pretest probability to identify caregivers needing help rather than applying the cumbersome questionnaires to all.

## Introduction

Caregiver strain is a well described entity that has been studied among different populations of caregivers for chronic medical conditions (psychiatric disorders, cancer, dementia, parkinsonism etc.) [1-4]. The physical, mental, social and spiritual well being of caregivers in these situations are continuously compromised (e.g. inability to attend to other family matters, frustration and anger towards self and patient, depression, financial issues, unemployment) [1,5,6]. Unless remedial action is taken, caretaker strain can have adverse effects on both the caregiver and the patient.

Data on caregiver strain in Sri Lanka is not available in published literature. In fact, most of the studies that are published refer to caregivers in developed nations [1,7,8]. The socio-economic milieu, support systems and level of medical care for patients differ vastly between these two groups of countries. Therefore the findings and recommendations cannot be applied directly to developing countries from the existing studies on the subject. In this light, it is important to look at the characteristics and patterns of caregiver strain in a developing country such as Sri Lanka to identify unique and specific problems that are in existence to work on more community tailored remedies.

## Methods

A prospective study was carried out in the university psychiatry unit of the National Hospital of Sri Lanka (NHSL) over a period of five weeks. All consenting principal caregivers of patients with schizophrenia and bipolar affective disorder were interviewed by CR. Background information such as demographics, socioeconomic standing, patients’ illness related data and adverse effects on the caregiver due to his/her role in patient care were extracted to a data sheet maintaining anonymity of the patient and the caregiver. The caregiver was also assessed with a screening tool for depression (Center for Epidemiological Studies – Depression scale, CES-D) [9], a brief quality of life assessment tool (Satisfaction with Life Scale, SWLS) [10] and a standardized tool for assessing care giver strain (Modified Caregiver Strain Index, CSI) [11].

The CES-D contains 20 questions and each has four answers with a common stem. Respective answers (in a question)are scored in ascending order (questions 4, 8, 12 and 16 scored in reverse order). Higher scores indicate a higher possibility of depression. The cut-offs in using the CES-D scores are as follows; less than 15; no depression, 16–21; mild depression, more than 21; possibility of severe depression [9]. SWLS contains five questions scored in a Likert scale (from 1 to 7). Higher scores show a better satisfaction with life. CSI has 13 questions on matters relating to caregiver strain in daily life that is scored as ‘yes – 2 points’, ‘no- 0 points’ and ‘sometimes- 1 point’. Higher scores indicate increased caregiver strain.

The data were analyzed with SPSS v15 statistical software, and significance of associations were calculated using chi square test/ Fishers exact test (for dichotomus data) and independent T test (for continuous data). Findings relevant to descriptive statistics were summarized into proportions and averages based on the scales of measurements. Ethical clearance for the study was granted by the Ethics Review Committee of the National Hospital of Sri Lanka.

## Results

Eighty caregivers were interviewed (males; 36, 45%). Their ages ranged from 21 to 84 years (mean: 57 years, SD: 13.3). Other demographic factors of caregivers are summarized in Table 1.

**Table 1:** Demographic characteristics of caregivers and illness related characteristics of their patients

**Characteristic**	**Number**	**Percentage (%)**
Gender		
*Male*	36	45.0
*Female*	44	55.0
Ethnicity		
*Sinhala*	77	96.3
*Tamil*	2	2.5
*Muslim*	1	1.3
Having permanent employment	32	40.0
Income* (Rs)		
*Less than 5000*	19	23.8
*5001 – 15,000*	26	32.5
*15,001 – 25,000*	20	25.0
*More than 25,000*	15	18.7
Level of education		
*No formal education*	3	3.8
*Primary education*	9	11.3
*Secondary education*	62	77.6
*Tertiary education*	6	7.6
Concurrent chronic medical problems**	37	46.3
Substance use		
*Alcohol*	18	22.5
*Nicotine*	12	15.0
*Other*		
Relationship to patient		
*Child*	33	41.2
*Parent*	11	13.7
*Sibling*	13	16.2
*Spouse*	10	12.5
*Other relation*	9	11.2
*Paid caregiver (non related)*	4	5.0
Illness		
*Schizophrenia*	65	81.3
*Bipolar affective disorder*	15	18.7
Disease activity		
*Active*	71	88.7
*In remission*	9	11.3
Duration of illness		
*Less than one year*	15	18.7
*1-5 years*	20	25.0
*More than 5 years*	45	56.3

* Conversion 1 USD = 130 rupees, ** Includes diabetes, hypertension, ischaemic heart disease, hypercholesterolaemia, heart failure, asthma and epilepsy.

The participants were asked about specific problems they had to face in caring for their patients and their responses are summarized in Table 2. Of the caregivers reporting assaults, only five (16.7%) reported frequent episodes (at least once a week). Of those in debt due to illness related issues (for consultation fees, medical care, institutionalization costs and fees for traditional healers), seven were having debts over Rs 50,000 (mean: Rs. 92,625, range: Rs. 7000 – 300,000). Almost half the caregivers (43.8%) admitted that they were under considerable psychological stress and requested professional help.

**Table 2:** Problems reported by caregivers that are related to their role of patient care

**Problem**	**Number**	**Percentage (%)**
Being subject to physical assault by patient	30	37.5
Being in debt due to illness related expenditures	19	23.8
Problems with employment including premature retirement, termination and resigning	17	21.3
Family problems with spouse (excluding patients)	24	30.0
Family problems with children (excluding patients)	27	33.8
Problems with other relations (excluding patients)	23	28.8
Disputes with neighbours	14	17.5
Having to give up significant interests including further studies and relationships	9	11.3
Increase in substance use	9	11.3
Self admission of needing professional help to overcome the mental stress	35	43.8

The results of the CES-D scale and the SWLS are summarized in Table 3. Overall, 30 (37.5%) caregivers screened positive for depression while 39 (48.8%) were below average on the life satisfaction scale. The overall mean CSI score for the sample was 7.95 (range: 0–21, SD: 5.8). A subgroup analysis with regard to depression, satisfaction with life and CSI scores are summarized in Table 4. Several factors including the amount of time spent with the patient, being in debt, conflicts with relations on patient related issues, being assaulted by patient on at least one occasion and interruption to work such as termination or early retirement were significantly associated with being depressed, being dissatisfied with life and having a higher caregiver strain. However, the most significant correlation was with the subjective feeling of being mentally strained (and self admission of needing professional help).

**Table 3:** Breakdown of CES-D scores and SWLS scores in the sample

**Scale and category**	**Number**	**Percentage (%)**
CES-D*		
*No depression*	50	62.5
*Possibility of mild depression*	21	26.2
*Possibility of severe depression*	9	11.3
SWLS		
*Highly satisfied*	11	13.8
*Satisfied*	9	11.3
*Average*	21	26.2
*Below average in life satisfaction*	20	25.0
*Dissatisfied*	17	21.3
*Highly dissatisfied*	2	2.5

**Table 4:** Comparison of caregiver subgroups against presence of depression, less than average satisfaction with life and caregiver strain index (CSI) scores

**Caregiver subgroup**	**Depression (n-30)vs.no depression (n-50)**	**Less than average SWLS (n-37) vs. others (n-43)**	**Comparison of CSI scores between subgroups**	
	Chi square value (df – 1)		Mean difference	95% confidence interval
Male vs. female	0.48	0.55	−0.92	−3.51 to 1.67
Level of education (Primary or no education vs. others)	0.94	7.81*	1.14	−2.47 to 4.75
Presence of a chronic medical problems	0.97	0.16	−1.62	−4.18 to 0.95
Smokers vs. non smokers	0.11	0.83	1.23	−2.37 to 4.84
Alcohol users vs. non users	0.17	0.51	−1.58	−4.66 to 1.49
Patient’s illness (schizophrenia vs. bipolar affective disorder)	5.0	6.36	1.37	−2.59 to 5.33
Duration of caregiver role (above sample average vs. below sample average)	0.18	0.08	0.841	−1.84 to 3.52
Symptoms of patient (active vs. remission)	2.37	0.27	2.44	−1.83 to 6.73
Relationship to patient (family member vs. other)	0.3	1.5	−0.98	−4.47 to 2.52
Time spent with patient per week (above sample average vs. below sample average )	9.37**	7.33**	4.6***	6.97 to 2.24
Being in debt	2.43	4.92*	5.8***	3.05 to 8.54
Employed vs. unemployed	0.01	0.13	0.24	−2.4 to 2.88
Having other dependents	0.01	0.25	−0.14	−2.73 to 2.44
Interruption to work	6.82*	1.37	4.25**	1.24 to 7.26
Subject to assault by patient	10.37**	3.65	4.56***	2.1 to 7.02
Having disputes with spouse	4.06	2.01	1.26	−1.54 to 4.07
Having disputes with children	5.67*	1.42	2.42	−0.25 to 5.1
Having disputes with other relations	7.52*	4.67*	3.61**	0.88 to 6.35
Having disputes with neighbours	1.13	0.08	1.32	−4.71 to 2.06
Self admission of needing professional help	21.13***	12.47***	5.11***	2.78 to 7.45

* p< 0.05, ** p < 0.01, *** p < 0.001.

## Discussion

Approximately 40% of the sample screened positive for depressive symptoms and 50% were dissatisfied with life circumstances. None of these caregivers so far has had the opportunity to have a formal therapy session for themselves as the primary focus was the patient. Yet, when specifically questioned 43% admitted that they also needed professional help to overcome their mental strain. This unidentified burden not only has a deleterious effect on caregivers but also on patients they are caring for. It also has an impact on the rest of the family if they are the sole breadwinners being responsible for the financial upkeep of the household.

The significant associations for being depressed among caregivers were, spending more time with the patient (above the group average), being assaulted by the patient, interruption to work, disputes with children and other relations and the subjective feeling of increased psychological burden. It is noted that other demographic factors that were thought to play a role such as level of education, amount of debt, disease characteristics of the patient and substance use of caregivers, were not associated with symptoms of depression. Adverse factors affecting the immediate environment of the caregiver and his/her emotional milieu on a daily basis were better predictors of depression. Conflicts with close family members, close association with the patient and being assaulted by the patient carry a heavier impact on the day-today mental status of the caregiver than the other issues mentioned above. It must also be noted that the caregiver may not be the sole breadwinner of the family and hence the effect of not being employed or being in debt may not be a direct concern. He or she may have adequate support from other family members and this will ‘dilute’ the impact of such financial adversities on the caregiver.

Lesser satisfaction with life was also associated with factors more likely to affect the caregiver’s immediate living environment (disputes with relations and spending more time with the patient). However being in debt and level of education was also significantly associated with having a low SWLS scores. The nature of questions asked in CES-D concentrates on the current situation in caregiver’s life. However, the questions in SWLS require the caregiver to look back and take a reflection of his/her entire life. Therefore factors such as being in debt (which is the net balance of life’s finances so far for a family or an individual) or inability to secure a better education and hence better opportunities in life may conspicuously stand out in SWLS scores.

The modified caregiver strain index assesses the extent of caregiver strain and like the CES-D concentrates more on the current disturbances to the caregiver’s life. Therefore the responses and significant associations observed paralleled those observed for CES-D scores, except the fact that being in debt, was a significant association for worse caregiver strain index scores.

We could not find any similar studies conducted in Sri Lanka in published literature. Literature on caregiver strain on schizophrenia and bipolar affective disorder is surprisingly sparse in the international indexing services but the conclusions from existing studies show this phenomenon to be of significant importance [12,13]. Some of our findings are in keeping with the findings of studies overseas while certain other observations are not. For example, it is a well established fact that interruption to work or termination of work significantly increases caregiver strain [14]. In many studies quoted in this article, gender of caregivers did not surface as a significant predictive factor of increased strain which was the same observation in our study. In a study of predictive validity of the resource deterioration model on recognizing depression among mothers of schizophrenic patients in the USA, coping resources and physical health of caregivers had value in the model but not social stressors like economic strain and burden of care [15]. Regarding contradictory findings, a study that assessed the caregivers’ state of health with the General Health Questionnaire (GHQ), concluded that the time spent with patient was irrelevant to the burden of care and mental strain of the caregiver. The strain of the caregivers’ marital relationship was more predictive of the mental strain [16].

Regarding the caregiver burden of bipolar affective disorder patients, the stress on caregivers on various aspects of patient behaviour (problem behaviours, role dysfunction and disturbance to household routine) was in the range of 50-80% in a study in the USA [17]. Those who had a higher burden were more likely to have depressive symptoms as well [18].

As mentioned previously, the caregiver strain is influenced by a variety of cultural and sociological modifiers that is specific to each community. It also depends on the support and coping strategies available to individuals. Therefore findings of one community cannot be applied to another directly. This is especially true when comparing developed and developing nations given the wide disparity of support services and treatment strategies available. This fact may explain certain disparities observed in our results and other studies of which the majority are from the USA. The key significance of this study is that we have identified several associations for depression and increased caregiver strain of principal caregivers of a subset of mentally ill patients in Sri Lanka that can be used to screen others in the same setting. This also adds to the very small number of papers available on the topic from developing countries. The most important point that stands out here is that the caregivers own discontent of their mental state is a significant factor associated with underlying depression or caregiver strain. Simply asking the question ‘do you think you are mentally run down and need professional help ?’ will be helpful in identifying a target population to screen (to increase pretest probability) rather than haphazardly applying the questionnaires in their entirety to all.

## Conclusions

This study assessed the symptoms of depression, satisfaction with life and caregiver strain of principal caregivers of patients with schizophrenia and bipolar affective disorder in Sri Lanka. The prevalence of symptoms of depression was 37.5%, while the percentage of those with unsatisfactory counts in SWLS was 48.8%. Presence of depression and having a higher caregiver strain was associated with spending more time with the patient, interruption to work, disputes with relations, being assaulted by patient and having a self admission to needing professional help to overcome the mental stress. The latter had the most significant correlation with depression, high CSI scores and a less than average satisfaction with life. These correlations need to be explored further with qualitative and quantitative studies to develop screening tools to identify those needing further assessment with standard questionnaires and a clinical interview.

## Competing interests

The authors have no competing interests to declare.

## Authors’ contributions

CR conceptualized the study. CR, VS and RH planned the design and proposal. RH and VH were responsible for clinical care of patients. CR and TF collected data. CR, TF and SR completed the analysis. All authors contributed to and approved the final manuscript.

## Authors’ information

CR is lecturer in Medicine and TF is a research assistant attached to the Department of Clinical Medicine, Faculty of Medicine, University of Colombo. SR is Professor in Medicine attached to the same department. RH and VS are consultant psychiatrists and senior lecturers attached to the Department of Psychological Medicine, Faculty of Medicine, University of Colombo.
